# *catena*-Poly[[(5,5′-dimethyl-2,2′-bi­pyridine)nickel(II)]-μ_2_-azido-κ^2^*N*:*N*-μ_2_-azido-κ^2^*N*:*N*′]: synthesis, crystal structure, Hirshfeld surface analysis and DFT calculations

**DOI:** 10.1107/S2056989025008151

**Published:** 2025-09-23

**Authors:** Zouaoui Setifi, Fatima Setifi, David K. Geiger, Thierry Maris, Loai Aljerf, Christopher Glidewell

**Affiliations:** a Département de Technologie Faculté de Technologie Université 20 Août 1955-Skikda BP 26 Route d'El-Hadaiek Skikda 21000 Algeria; b Laboratoire de Chimie Ingénierie Moléculaire et Nanostructures (LCIMN) Université Ferhat Abbas Sétif 1 Sétif 19000 Algeria; c Department of Chemistry, SUNY-College at Geneseo, Geneseo, NY 14454, USA; d Department of Chemistry, Université de Montréal, Campus MIL, 1375 Avenue Thérèse Lavoie-Roux, Montréal (Québec) H2V 0B3, Canada; e Laboratory of Organic Industries, Department of Chemistry, Faculty of Sciences, Damascus University, Damascus, Syrian Arab Republic; f School of Chemistry, University of St Andrews, St Andrews, Fife KY16 9ST, United Kingdom; Tokyo University of Science, Japan

**Keywords:** solvothermal synthesis, nickel complex, azido ligand, crystal structure, coordination polymer, Hirshfeld surface analysis, DFT calculations

## Abstract

The title compound, [Ni(N_3_)_2_(C_12_H_12_N_2_)]_*n*_, was synthesized solvothermally and characterized crystallographically. The compound forms a one-dimensional coordination polymer containing alternating spiro-fused four- and eight-membered rings, in which both ring types are centrosymmetric. Adjacent polymer chains are linked into sheets by means of a single C—H⋯N hydrogen bond. Hirshfeld surface analysis was used to investigate the inter­molecular inter­actions. DFT calculations were used to explore the conformation of the bridging azides and to compare the stability of this compound with those previously reported [Setifi *et al.* (2022 ▸ ▸). *Acta Cryst*. C**78**, 449–454].

## Abstract 2

The title compound, [Ni(N_3_)_2_(C_12_H_12_N_2_)]_*n*_, was synthesized solvothermally and characterized crystallographically. In the crystal, adjacent polymer chains are linked into sheets by means of a single C—H⋯N hydrogen bond.

## 1. Chemical context

Coordination polymers (CPs) have received significant attention due to their inter­esting and diverse topologies, and potential applications in various fields including magnetism (Setifi *et al.*, 2009 ▸; Yuste *et al.*, 2009 ▸; Merabet *et al.*, 2022 ▸; He *et al.*, 2018 ▸). Various coordination polymers from 1D to 3D networks along with their magnetic studies have been reported (Atmani *et al.*, 2008 ▸; Benmansour *et al.*, 2008 ▸, 2010 ▸). The structural flexibility and electronic characteristics of the organic ligand, and also the nature of the metal ion are important factors for the construction of CPs (He *et al.*, 2014 ▸). In addition, the dimensionality of CPs could be enhanced by selection of suitable linkers. Cyano­carbanions and pseudo­halides are inter­esting bridging ligands because of their structural versatility in coordination chemistry (Benmansour *et al.*, 2007 ▸; Mautner *et al.*, 2019 ▸; Dmitrienko *et al.*, 2020 ▸). In particular, the azide ion is a highly symmetric anion that has small size and linear shape. Hence, it has a high ability to propagate magnetic inter­actions between paramagnetic centres, leading to CPs with inter­esting magnetic properties (Setifi *et al.*, 2016 ▸; Setifi, Setifi *et al.*, 2022 ▸). Additionally, the coordinated azide has two N—N bonds, which are less symmetric compared to the free one where the degree of asymmetry depends on the azide bonding mode. Among the pool of bonding modes of azide ions, the end-on (EO) and end-to-end (EE) are the most prevalent. These bridging modes are responsible for the construction of coordination compounds with varying nuclearity and dimensionality (Benamara *et al.*, 2021 ▸; Merabet *et al.*, 2023 ▸).
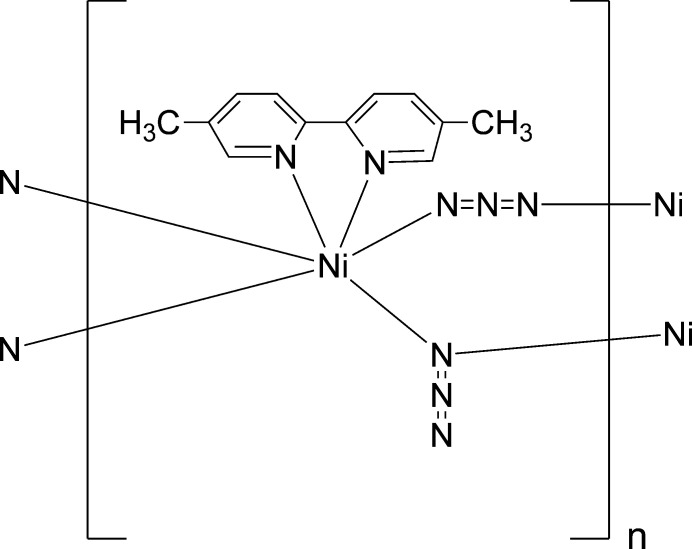


In the light of the exciting coordination chemistry of the azide ion as a linker, the current work aimed to synthesize new azido CPs comprising 5,5′-dimethyl-2,2′-di­pyridine as co-ligand and the azide anion as ligand. The crystal and mol­ecular structures of the title compound (I) are described, a study complemented by an analysis of the mol­ecular packing by calculating the Hirshfeld surfaces as well as a computational chemistry study.

## 2. Structural commentary

**Figure 1:**
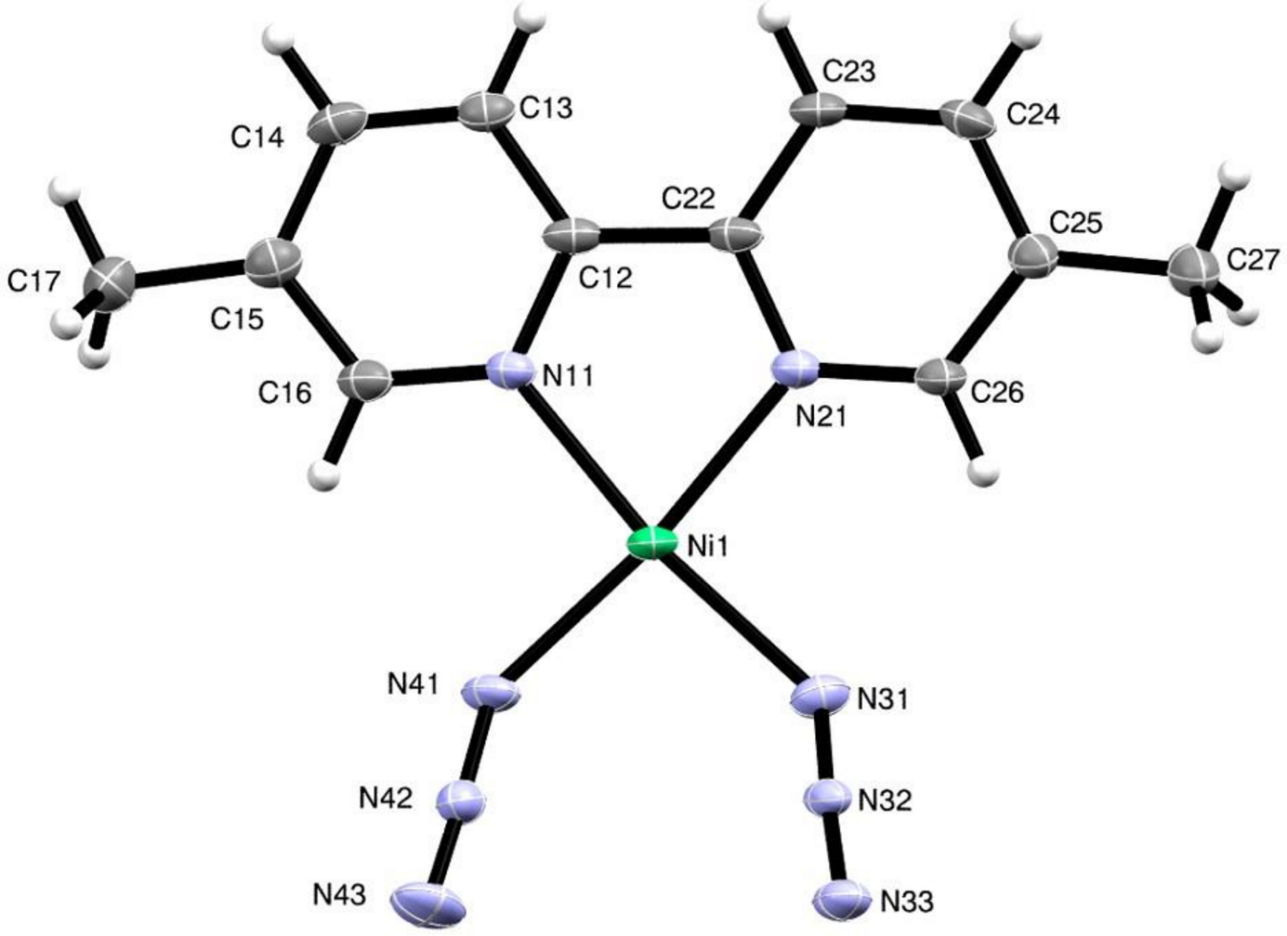
The selected asymmetric unit in compound (I) showing the atom-labelling scheme. Displacement ellipsoids are drawn at the 30% probability level.

**Figure 2:**
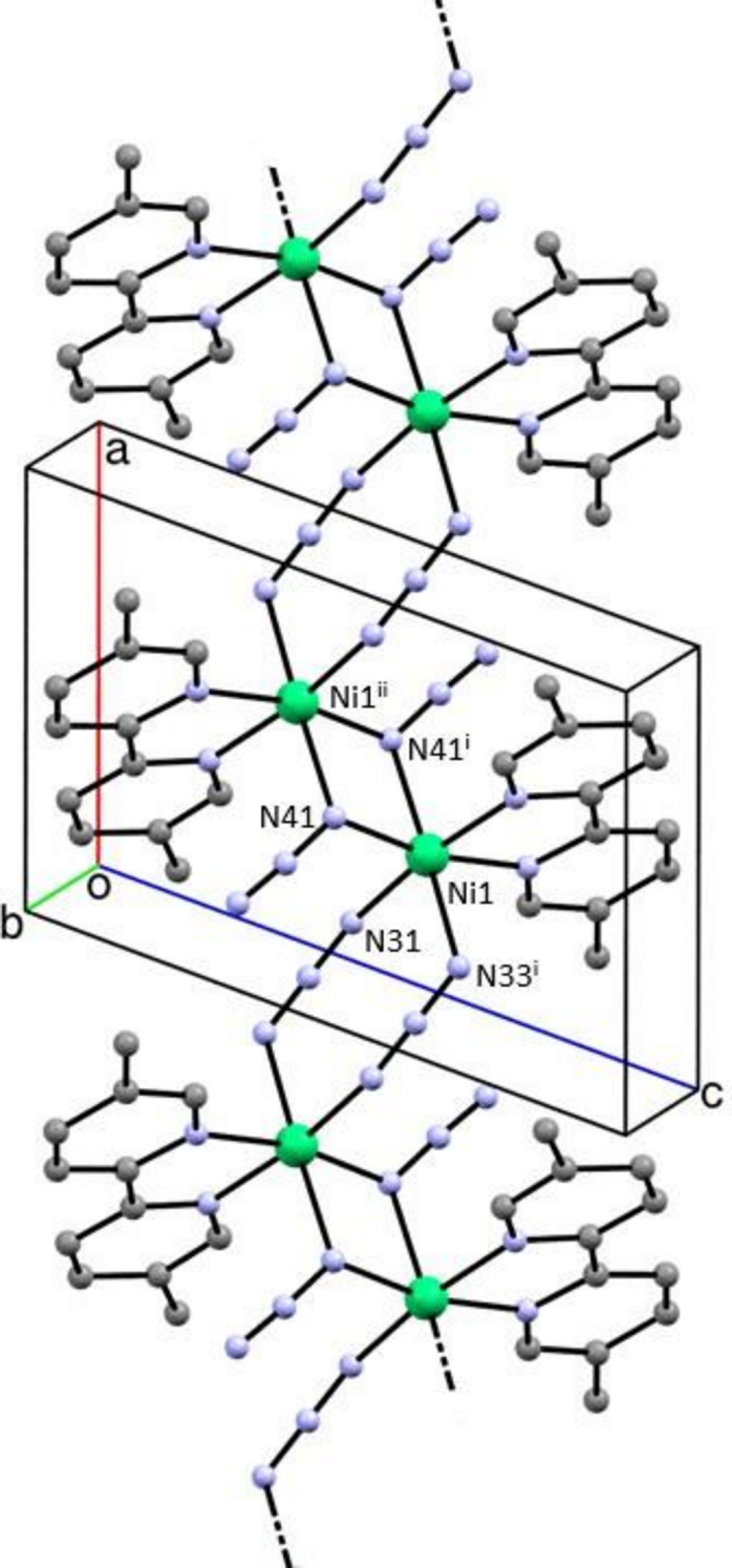
Part of the crystal structure of compound (I) showing the formation of a chain of alternating four- and eight-membered rings running parallel to the [100] direction. For the sake of clarity, the H atoms have all been omitted. For symmetry codes, see Tables 1 ▸–3 ▸ ▸.

The structure consists of a bidentate 5,5′-dimethyl-2,2′-di­pyridine ligand coordinated to the nickel(II) centre along with two azido ligands (Fig. 1 ▸). Each of the anionic ligands acts a bridge between two metal centres, but they act in different ways. For one of the azido ligands, the two terminal atoms N31 and N33 (Fig. 1 ▸) coordinate to two inversion-related Ni centres, so forming a centrosymmetric eight-membered ring (Fig. 2 ▸). In the other azido ligand, one of the terminal atoms, N41, coordinates to a different pair of inversion-related Ni centres, so forming a centrosymmetric four-membered ring (Fig. 2 ▸), but the other terminal atom, N43, plays no part in the coordination. The resulting polymeric structure is thus a chain of spiro-fused rings running parallel to the [100] direction, in which four-membered rings centred at (*n* + 0.5, 0.5, 0.5) alternate with eight-membered rings centred at (*n*, 0.5, 0.5), where *n* represents an integer in each case (Fig. 2 ▸). Regardless of the coordination modes, the N—N distances (Table 1 ▸) are all closely grouped within the range 2.045 (5) to 2.171 (7) Å and the N—N distances within the two independent azido ligands are all quite similar. Within the di­pyridine ligand, the two independent rings are not quite coplanar, the dihedral angle between the rings being 3.9 (3) °.

Within the selected asymmetric unit (Fig. 1 ▸), the metallacyclic ring is effectively planar, while the centrosymmetric four-membered ring in the coordination polymer is necessarily planar. The eight-membered ring in the polymer adopts a chair form: the six N atoms within this ring are almost coplanar, with an r.m.s. deviation from the mean plane of only 0.100 Å, but the inversion-related Ni atoms are displaced from this plane by 0.702 (10) Å.

## 3. Supra­molecular features

**Figure 3:**
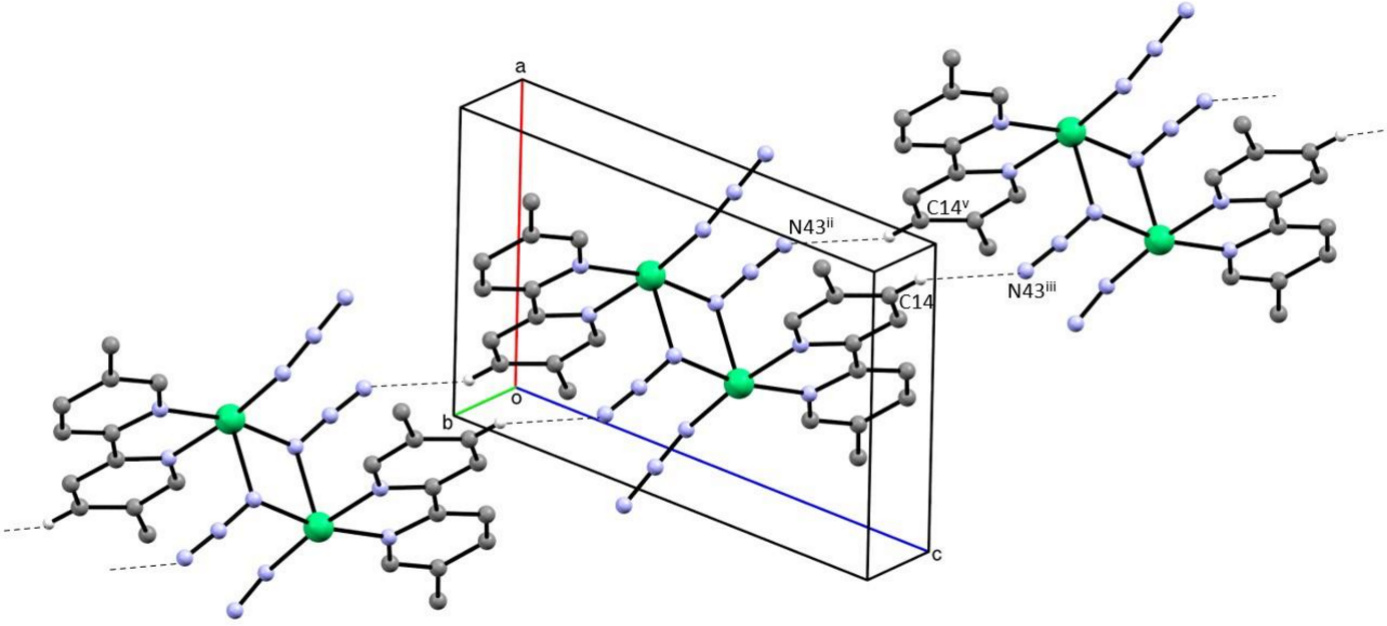
Part of the crystal structure of compound (I) showing the formation of a chain of rings running parallel to the [101] direction. Hydrogen bonds are drawn as dashed lines, and the H atoms which are not involved in the motif shown have been omitted. For symmetry codes, see Tables 2 ▸–4 ▸ ▸.

**Figure 4:**
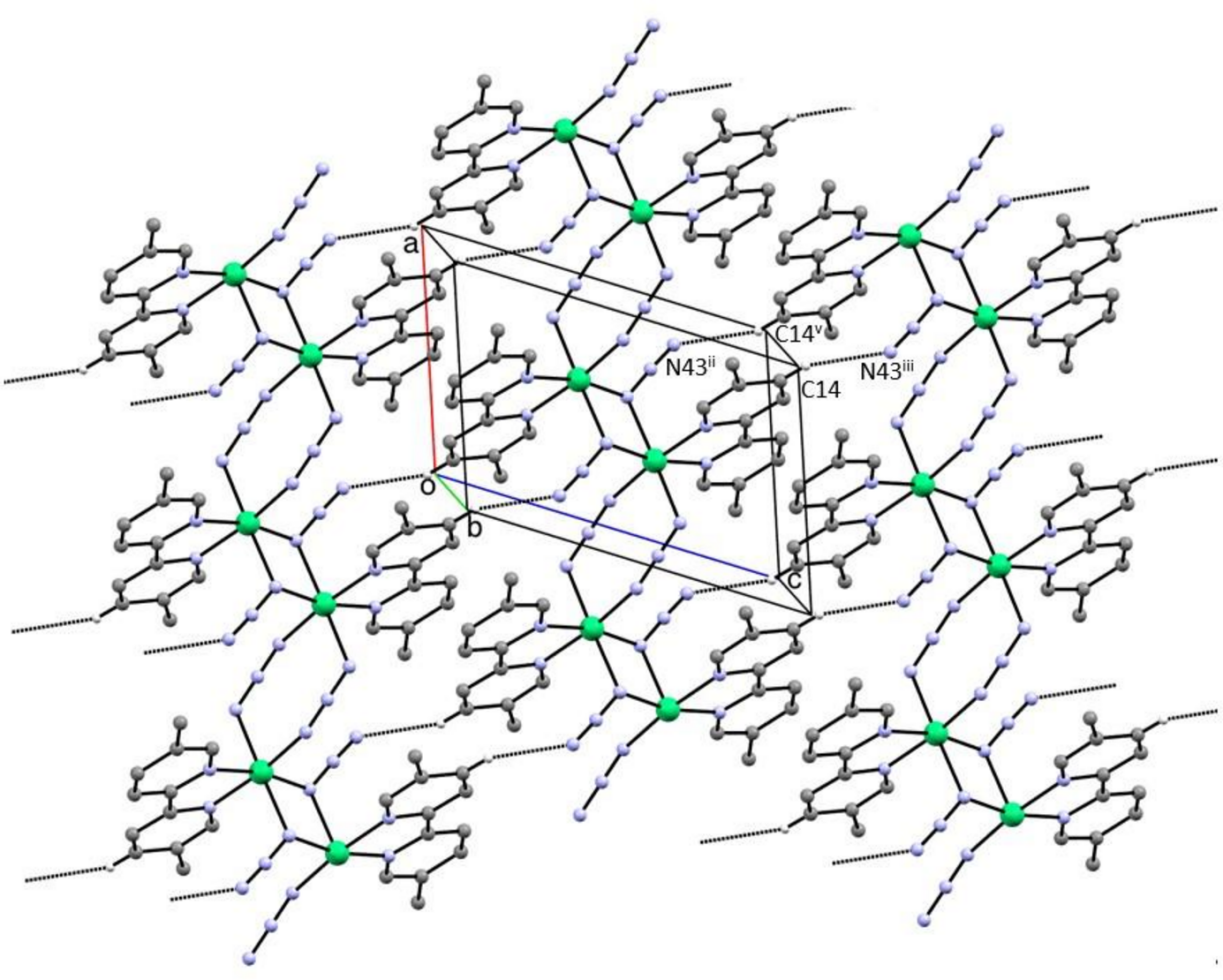
Part of the crystal structure of compound (I) showing the formation of a sheet parallel to (010) formed by hydrogen bonding between adjacent coordination polymer chains. Hydrogen bonds are drawn as dashed lines, and the H atoms which are not involved in the motif shown have been omitted. For symmetry codes (i) to (iv), see Tables 1 ▸–3 ▸ ▸. Symmetry code: (v) 2 − *x*, 1 − *y*, 2 − *z*.

There is only one significant hydrogen bond in the structure of compound (I) (Table 2 ▸), and this gives rise to a chain of rings running parallel to the [101] direction (Fig. 3 ▸). Within this chain, hydrogen bonded rings of the 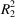
(18) type (Etter, 1990 ▸; Etter *et al.*, 1990 ▸; Bernstein *et al.*, 1995 ▸) are centred at (*n*, 0.5, *n*), where *n* represents an integer, and these rings alternate with four-membered metallacyclic rings centred at (*n* + 0.5, 0.5, *n* + 0.5), where *n* again represents an integer. The combination of chains of this type parallel to [101] and the coordination polymer chains parallel to [100] gives rise to a complex sheet lying parallel to (001), where the reference chain lies in the domain 0.25 < *y* < 0.75 (Fig. 4 ▸). A second sheet, related to the reference sheet by the translational symmetry elements, lies in the domain 0.75 < *y* < 1.25.

The only other short C—H⋯N contact involves one of the methyl groups (Table 2 ▸), but this contact is unlikely to be of structural significance, not only because methyl C—H bonds are of low acidity, but because such methyl groups are usually undergoing very rapid rotation about the adjacent C—C bond (Riddell & Rogerson, 1996 ▸, 1997 ▸), while sixfold rotational barriers, of the type found in methyl arenes, are particularly small (Tannenbaum *et al.*, 1956 ▸; Naylor & Wilson, 1957 ▸).

There is a single anion⋯π inter­action (Table 3 ▸), which lies within the reference coordination polymer chain; there is also a short π–π contact [centroid–centroid distance = 3.889 ’(3) Å] lying in the hydrogen-bonded sheet. Thus neither of these inter­actions has any effect on the overall dimensionality of the supra­molecular assembly.

## 4. Database survey

A search of the Cambridge Structural Database [Version 6.00 with one update (August 2025); Groom *et al.*, 2016 ▸] returned about 162 structures with nickel linked to at least one azido ligand. Among them, 31 structures have an eight-membered ring as found in the title compound, while the four-membered rings involving a pendant azido ligand is found in 98 structures. Two structures only involve both rings: HISLEB (Song *et al.*, 2007 ▸) and ZIJFUT01 (Monfort *et al.*, 2000 ▸). A search for compounds with nickel linked to 5,5′-dimethyl-2,2′-bi­pyridine ligand returned 26 hits. Only two structures include both the azido ion and the 5,5′-dimethyl-2,2′-bi­pyridine ligand, MUBWEM (Phatchimkun *et al.*, 2009 ▸) and POMFAZ (Hou *et al.*, 2008 ▸), the later featuring a four-membered ring.

## 5. Hirshfeld surface analysis and inter­action energy calculations

The Hirshfeld surface analyses were performed using the program *CrystalExplorer17* (Spackman *et al.*, 2021 ▸; Turner *et al.*, 2017 ▸). All energy calculations were performed on mol­ecules in the gas phase using *SPARTAN’20* (Wavefunction, 2020 ▸). DFT calculations using the M06-2X (Zhao & Truhlar, 2008 ▸) functionals with a 6-31G(d,p) basis set were employed. Atomic coordinates obtained from the crystallographic analysis were used for all non-H atoms. As the bond lengths obtained for H atoms from X-ray crystallographic analyses are inaccurate, the positions of the H atoms were adjusted based on normalized values determined by neutron diffraction results.

**Figure 5:**
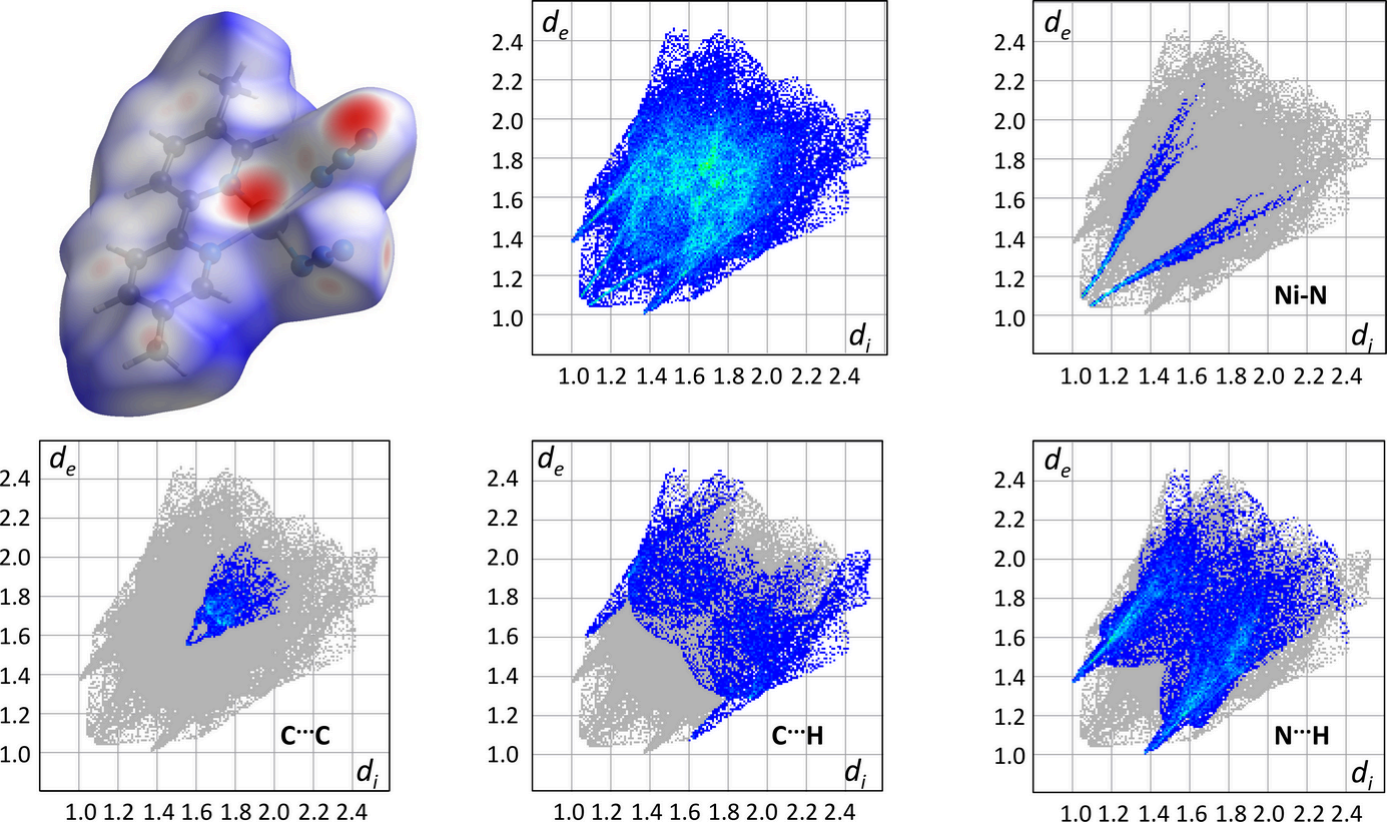
Hirshfeld surface and fingerprint plots.

The Hirshfeld surface and fingerprint plots are displayed in Fig. 5 ▸. Calculations were performed on the asymmetric unit. The closeness of inter­actions is indicated on a diminishing scale from red to blue. Thus, the reddest regions of the surface correspond to the Ni—N(azide) bonds (see the Ni–N inset in Fig. 5 ▸). The weak π–π inter­action is responsible for the blue patch observed in the C⋯C inset. Finally, the C—H⋯N hydrogen bond is represented in the N⋯H inset. Surface coverage corresponds to 31.9% H⋯H, 14.5% C⋯H, 31.0% N⋯H, 9.9% N⋯N and 4.3% C⋯C.

We recently reported the structures of dimorphic forms of an iron(II) complex (Setifi, Bernès *et al.*, 2022 ▸). A significant structural difference between the two forms (one *P*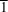 and the other *P*2_1_/*c*) is the conformation of the eight-membered Fe(μ-1,3-N_3_)_2_ ring. Although the ring in both structures exhibits a chair conformation, the angle δ, defined as the angle formed by the (N_3_)_2_ mean plane and the plane formed by the metal and the bonded N(azide) atoms, is 8.2° in the triclinic form and 25.6° in the monoclinic form. DFT calculations revealed that the monoclinic form is more stable than the triclinic form by *ca* 30.5 kJ mol^−1^. In the nickel(II) complex, δ = 27.6°. DFT calculations show that this structure is *ca* 20.83 kJ mol^−1^ more stable than a hypothetical structure in which δ is reduced to 8.2°.

## 6. Synthesis and crystallization

A mixture of nickel(II) nitrate hexa­hydrate (58 mg, 0.2 mmol), 5,5′-dimethyl-2,2′-bi­pyridine (37 mg, 0.2 mmol), sodium azide (26 mg, 0.4 mmol), N,N-di­methyl­formamide (12 ml) and water (6 ml) was sonicated for 30 min. Then the reaction mixture was transferred to a Teflon-lined stainless steel reactor and placed in the oven. Subsequently, the temperature was kept 403 K for 2 days. After cooling to room temperature at a rate of 10 Kh^−1^, green block-shaped crystals of (I) were obtained.

## 7. Refinement

Crystal data, data collection and refinement details are summarized in Table 4 ▸. The refinement was handled as a two-component twin, with twin matrix (1,0,0/0,-1,0/-1,0,-1) and with refined twin fractions 0.486 (3) and 0.514 (3). All H atoms were located in difference maps and then treated as riding atoms in geometrically idealized positions with C—H distances of 0.95 Å (pyridine) or 0.98 Å (methyl) and with *U*_iso_(H) = *kU*_eq_(C), where *k* = 1.5 for the methyl groups, which were permitted to rotate but not to tilt, and 1.2 for the pyridine H atoms.

## Supplementary Material

Crystal structure: contains datablock(s) global, I. DOI: 10.1107/S2056989025008151/jp2019sup1.cif

Structure factors: contains datablock(s) I. DOI: 10.1107/S2056989025008151/jp2019Isup2.hkl

CCDC reference: 2487940

Additional supporting information:  crystallographic information; 3D view; checkCIF report

## Figures and Tables

**Table 1:** Selected bond lengths (Å)

Ni1—N11	2.045 (5)	Ni1—N41^ii^	2.165 (6)
Ni1—N21	2.074 (5)	N31—N32	1.167 (7)
Ni1—N31	2.096 (5)	N32—N33	1.175 (8)
Ni1—N41	2.090 (6)	N41—N42	1.217 (7)
Ni1—N33^i^	2.171 (7)	N42—N43	1.141 (8)

Symmetry codes: (i) 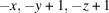; (ii) 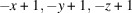.

**Table 2:** Hydrogen-bond and short intermolecular contact parameters (Å, °)

*D*—H⋯*A*	*D*—H	H⋯*A*	*D*⋯*A*	*D*—H⋯*A*
C14—H14⋯N43^iii^	0.95	2.50	3.334 (9)	147
C17—H17*C*⋯N43^iv^	0.98	2.55	3.492 (11)	162

Symmetry codes: (iii) 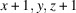; (iv) 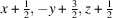.

**Table 3:** Parameters (Å, °) for the short anion⋯π contact *Cg*1 represents the centroid of the N21/C22–C26 ring.

N42—N43⋯*Cg*1	N42—N43	N43⋯*Cg*	N42⋯*Cg*	N42—N43⋯*Cg*
N42—N43⋯*Cg*1^ii^	1.141 (8)	3.967 (8)	3.917 (6)	79.2 (5)

Symmetry code: (ii) 1 − *x*, 1 − *y*, 1 − *z*.

**Table 4:** Experimental details

Crystal data	
Chemical formula	[Ni(N_3_)_2_(C_12_H_12_N_2_)]
*M*_r_	327.01
Crystal system, space group	Monoclinic, *P*2_1_/*n*
Temperature (K)	150
*a*, *b*, *c* (Å)	7.2641 (9), 18.395 (2), 10.5001 (12)
β (°)	110.223 (7)
*V* (Å^3^)	1316.6 (3)
*Z*	4
Radiation type	Ga *K*α, λ = 1.34139 Å
μ (mm^−1^)	8.09
Crystal size (mm)	0.25 × 0.10 × 0.03

Data collection	
Diffractometer	Bruker Venture Metaljet
Absorption correction	Multi-scan (*SADABS*; Krause *et al.*, 2015 ▸)
*T*_min_, *T*_max_	0.415, 0.752
No. of measured, independent and observed [*I* > 2σ(*I*)] reflections	14498, 2532, 2381
*R*_int_	0.0741
(sin θ/λ)_max_ (Å^−1^)	0.611

Refinement	
*R*[*F*^2^ > 2σ(*F*^2^)], *wR*(*F*^2^), *S*	0.063, 0.167, 1.08
No. of reflections	2532
No. of parameters	193
H-atom treatment	H-atom parameters constrained
Δρ_max_, Δρ_min_ (e Å^−3^)	1.20, −0.60

Computer programs: *APEX2* (Bruker, 2013 ▸), *SAINT* (Bruker, 2017 ▸), *SHELXT* (Sheldrick, 2015*a* ▸), *SHELXL2014* (Sheldrick, 2015*b* ▸) and *PLATON* (Spek, 2020 ▸).

## supplementary crystallographic information

### catena-Poly[[(5,5'-dimethyl-2,2'-bipyridine)nickel(II)]-µ2-azido-κ2N:N-µ2-azido-κ2N:N'] Crystal data

**Table:**

[Ni(N_3_)_2_(C_12_H_12_N_2_)]	*F*(000) = 672
*M**_r_* = 327.01	*D*_x_ = 1.650 Mg m^−^^3^
Monoclinic, *P*2_1_/*n*	Ga *K*α radiation, λ = 1.34139 Å
*a* = 7.2641 (9) Å	Cell parameters from 3083 reflections
*b* = 18.395 (2) Å	θ = 4.2–61.0°
*c* = 10.5001 (12) Å	µ = 8.09 mm^−^^1^
β = 110.223 (7)°	*T* = 150 K
*V* = 1316.6 (3) Å^3^	Block, green
*Z* = 4	0.25 × 0.10 × 0.03 mm

### catena-Poly[[(5,5'-dimethyl-2,2'-bipyridine)nickel(II)]-µ2-azido-κ2N:N-µ2-azido-κ2N:N'] Data collection

**Table:**

Bruker Venture Metaljet diffractometer	2532 independent reflections
Radiation source: Gallium Liquid Metal Jet Source	2381 reflections with *I* > 2σ(*I*)
Helios MX Mirror Optics monochromator	*R*_int_ = 0.074
φ and ω scans	θ_max_ = 55.0°, θ_min_ = 4.2°
Absorption correction: multi-scan (SADABS; Krause *et al.*, 2015)	*h* = −8→8
*T*_min_ = 0.415, *T*_max_ = 0.752	*k* = −22→22
14498 measured reflections	*l* = −12→12

### catena-Poly[[(5,5'-dimethyl-2,2'-bipyridine)nickel(II)]-µ2-azido-κ2N:N-µ2-azido-κ2N:N'] Refinement

**Table:**

Refinement on *F*^2^	Primary atom site location: difference Fourier map
Least-squares matrix: full	Hydrogen site location: inferred from neighbouring sites
*R*[*F*^2^ > 2σ(*F*^2^)] = 0.063	H-atom parameters constrained
*wR*(*F*^2^) = 0.167	*w* = 1/[σ^2^(*F*_o_^2^) + (0.0872*P*)^2^ + 3.9821*P*] where *P* = (*F*_o_^2^ + 2*F*_c_^2^)/3
*S* = 1.08	(Δ/σ)_max_ < 0.001
2532 reflections	Δρ_max_ = 1.20 e Å^−^^3^
193 parameters	Δρ_min_ = −0.60 e Å^−^^3^
0 restraints	

### catena-Poly[[(5,5'-dimethyl-2,2'-bipyridine)nickel(II)]-µ2-azido-κ2N:N-µ2-azido-κ2N:N'] Special details

**Table:**

Geometry. All esds (except the esd in the dihedral angle between two l.s. planes) are estimated using the full covariance matrix. The cell esds are taken into account individually in the estimation of esds in distances, angles and torsion angles; correlations between esds in cell parameters are only used when they are defined by crystal symmetry. An approximate (isotropic) treatment of cell esds is used for estimating esds involving l.s. planes.
Refinement. Refined as a 2-component twin.

### catena-Poly[[(5,5'-dimethyl-2,2'-bipyridine)nickel(II)]-µ2-azido-κ2N:N-µ2-azido-κ2N:N'] Fractional atomic coordinates and isotropic or equivalent isotropic displacement parameters (Å^2^)

**Table:**

	*x*	*y*	*z*	*U*_iso_*/*U*_eq_	
Ni1	0.38091 (13)	0.48792 (5)	0.60721 (8)	0.0330 (3)	
N11	0.5941 (8)	0.5401 (3)	0.7604 (5)	0.0313 (11)	
C12	0.6594 (8)	0.5047 (4)	0.8810 (6)	0.0330 (14)	
C13	0.8008 (9)	0.5363 (4)	0.9928 (6)	0.0333 (13)	
H13	0.8445	0.5117	1.0776	0.040*	
C14	0.8764 (9)	0.6028 (4)	0.9801 (6)	0.0390 (15)	
H14	0.9721	0.6247	1.0565	0.047*	
C15	0.8130 (10)	0.6389 (4)	0.8544 (7)	0.0400 (16)	
C16	0.6708 (10)	0.6051 (4)	0.7497 (6)	0.0364 (14)	
H16	0.6239	0.6291	0.6644	0.044*	
N21	0.4256 (7)	0.4145 (3)	0.7650 (5)	0.0307 (11)	
C22	0.5683 (9)	0.4329 (3)	0.8828 (6)	0.0322 (13)	
C23	0.6190 (9)	0.3871 (4)	0.9939 (6)	0.0364 (15)	
H23	0.7206	0.4006	1.0755	0.044*	
C24	0.5233 (10)	0.3222 (4)	0.9869 (6)	0.0385 (15)	
H24	0.5608	0.2903	1.0627	0.046*	
C25	0.3711 (10)	0.3031 (3)	0.8687 (6)	0.0368 (14)	
C26	0.3320 (10)	0.3517 (3)	0.7603 (6)	0.0344 (14)	
H26	0.2318	0.3391	0.6774	0.041*	
C17	0.8958 (12)	0.7117 (4)	0.8324 (8)	0.0517 (19)	
H17A	0.9306	0.7095	0.7503	0.077*	
H17B	1.0131	0.7229	0.9107	0.077*	
H17C	0.7972	0.7497	0.8221	0.077*	
C27	0.2533 (13)	0.2357 (4)	0.8602 (7)	0.0492 (18)	
H27A	0.2860	0.2002	0.8017	0.074*	
H27B	0.1133	0.2475	0.8220	0.074*	
H27C	0.2832	0.2151	0.9512	0.074*	
N31	0.1551 (8)	0.4250 (3)	0.4743 (5)	0.0380 (12)	
N32	−0.0029 (8)	0.4410 (3)	0.4041 (5)	0.0332 (11)	
N33	−0.1640 (9)	0.4538 (3)	0.3329 (5)	0.0426 (14)	
N41	0.3989 (8)	0.5617 (3)	0.4606 (5)	0.0385 (12)	
N42	0.2591 (8)	0.5944 (3)	0.3833 (5)	0.0375 (12)	
N43	0.1340 (9)	0.6266 (4)	0.3084 (7)	0.0562 (18)	

### catena-Poly[[(5,5'-dimethyl-2,2'-bipyridine)nickel(II)]-µ2-azido-κ2N:N-µ2-azido-κ2N:N'] Atomic displacement parameters (Å^2^)

**Table:**

	*U*^11^	*U*^22^	*U*^33^	*U*^12^	*U*^13^	*U*^23^
Ni1	0.0330 (5)	0.0389 (4)	0.0192 (4)	−0.0003 (4)	−0.0008 (4)	0.0018 (4)
N11	0.032 (3)	0.036 (3)	0.023 (2)	0.004 (2)	0.005 (2)	0.002 (2)
C12	0.025 (3)	0.051 (4)	0.021 (3)	0.009 (2)	0.005 (2)	0.001 (3)
C13	0.031 (3)	0.045 (3)	0.024 (3)	0.005 (3)	0.009 (2)	−0.003 (2)
C14	0.028 (3)	0.050 (4)	0.032 (3)	0.006 (3)	0.002 (3)	−0.008 (3)
C15	0.033 (3)	0.047 (4)	0.039 (4)	0.002 (3)	0.012 (3)	−0.002 (3)
C16	0.037 (3)	0.040 (3)	0.027 (3)	0.007 (3)	0.004 (3)	0.000 (3)
N21	0.029 (2)	0.037 (3)	0.023 (2)	0.002 (2)	0.005 (2)	0.002 (2)
C22	0.032 (3)	0.038 (3)	0.021 (3)	0.014 (2)	0.003 (2)	−0.001 (2)
C23	0.033 (3)	0.049 (4)	0.020 (3)	0.005 (3)	0.001 (2)	0.002 (3)
C24	0.045 (4)	0.041 (4)	0.026 (3)	0.012 (3)	0.007 (3)	0.010 (3)
C25	0.045 (4)	0.035 (3)	0.029 (3)	0.002 (3)	0.011 (3)	−0.001 (2)
C26	0.038 (3)	0.039 (3)	0.021 (3)	0.000 (3)	0.004 (3)	0.001 (2)
C17	0.049 (4)	0.049 (4)	0.050 (4)	−0.010 (3)	0.008 (3)	−0.002 (3)
C27	0.067 (5)	0.038 (3)	0.039 (4)	−0.002 (4)	0.013 (4)	0.000 (3)
N31	0.027 (3)	0.053 (3)	0.030 (3)	0.002 (3)	0.005 (3)	−0.003 (2)
N32	0.040 (3)	0.030 (2)	0.022 (2)	0.003 (2)	0.002 (3)	−0.0008 (19)
N33	0.041 (3)	0.045 (3)	0.030 (3)	0.006 (3)	−0.003 (3)	0.002 (2)
N41	0.037 (3)	0.043 (3)	0.026 (2)	0.010 (3)	−0.001 (2)	0.004 (2)
N42	0.036 (3)	0.041 (3)	0.030 (2)	−0.006 (3)	0.005 (3)	0.003 (2)
N43	0.036 (3)	0.077 (5)	0.045 (3)	0.017 (3)	0.001 (3)	0.021 (3)

### catena-Poly[[(5,5'-dimethyl-2,2'-bipyridine)nickel(II)]-µ2-azido-κ2N:N-µ2-azido-κ2N:N'] Geometric parameters (Å, º)

**Table:**

Ni1—N11	2.045 (5)	C23—C24	1.370 (10)
Ni1—N21	2.074 (5)	C23—H23	0.9500
Ni1—N31	2.096 (5)	C24—C25	1.392 (9)
Ni1—N41	2.090 (6)	C24—H24	0.9500
Ni1—N33^i^	2.171 (7)	C25—C26	1.397 (9)
Ni1—N41^ii^	2.165 (6)	C25—C27	1.491 (10)
N11—C16	1.340 (9)	C26—H26	0.9500
N11—C12	1.354 (7)	C17—H17A	0.9800
C12—C13	1.392 (8)	C17—H17B	0.9800
C12—C22	1.482 (9)	C17—H17C	0.9800
C13—C14	1.366 (10)	C27—H27A	0.9800
C13—H13	0.9500	C27—H27B	0.9800
C14—C15	1.406 (9)	C27—H27C	0.9800
C14—H14	0.9500	N31—N32	1.167 (7)
C15—C16	1.371 (9)	N32—N33	1.175 (8)
C15—C17	1.517 (11)	N33—Ni1^i^	2.171 (7)
C16—H16	0.9500	N41—N42	1.217 (7)
N21—C26	1.332 (8)	N41—Ni1^ii^	2.165 (6)
N21—C22	1.354 (7)	N42—N43	1.141 (8)
C22—C23	1.382 (8)		
			
N11—Ni1—N21	79.1 (2)	C22—N21—Ni1	115.2 (4)
N11—Ni1—N41	93.0 (2)	N21—C22—C23	121.1 (6)
N21—Ni1—N41	168.2 (2)	N21—C22—C12	114.5 (5)
N11—Ni1—N31	171.1 (2)	C23—C22—C12	124.4 (5)
N21—Ni1—N31	92.4 (2)	C24—C23—C22	120.2 (6)
N41—Ni1—N31	95.8 (2)	C24—C23—H23	119.9
N11—Ni1—N41^ii^	90.7 (2)	C22—C23—H23	119.9
N21—Ni1—N41^ii^	93.4 (2)	C23—C24—C25	120.0 (6)
N41—Ni1—N41^ii^	77.9 (2)	C23—C24—H24	120.0
N31—Ni1—N41^ii^	92.6 (2)	C25—C24—H24	120.0
N11—Ni1—N33^i^	88.2 (2)	C24—C25—C26	116.1 (6)
N21—Ni1—N33^i^	91.1 (2)	C24—C25—C27	121.7 (6)
N41—Ni1—N33^i^	97.4 (2)	C26—C25—C27	122.1 (6)
N31—Ni1—N33^i^	89.2 (2)	N21—C26—C25	124.5 (6)
N41^ii^—Ni1—N33^i^	175.1 (2)	N21—C26—H26	117.7
C16—N11—C12	119.0 (5)	C25—C26—H26	117.7
C16—N11—Ni1	125.2 (4)	C15—C17—H17A	109.5
C12—N11—Ni1	115.8 (4)	C15—C17—H17B	109.5
N11—C12—C13	120.4 (6)	H17A—C17—H17B	109.5
N11—C12—C22	115.2 (5)	C15—C17—H17C	109.5
C13—C12—C22	124.3 (5)	H17A—C17—H17C	109.5
C14—C13—C12	119.7 (6)	H17B—C17—H17C	109.5
C14—C13—H13	120.1	C25—C27—H27A	109.5
C12—C13—H13	120.1	C25—C27—H27B	109.5
C13—C14—C15	120.2 (6)	H27A—C27—H27B	109.5
C13—C14—H14	119.9	C25—C27—H27C	109.5
C15—C14—H14	119.9	H27A—C27—H27C	109.5
C16—C15—C14	116.7 (6)	H27B—C27—H27C	109.5
C16—C15—C17	120.5 (6)	N32—N31—Ni1	130.8 (5)
C14—C15—C17	122.8 (6)	N31—N32—N33	177.0 (7)
N11—C16—C15	123.9 (6)	N32—N33—Ni1^i^	125.3 (5)
N11—C16—H16	118.0	N42—N41—Ni1	124.2 (5)
C15—C16—H16	118.0	N42—N41—Ni1^ii^	122.0 (4)
C26—N21—C22	118.0 (5)	Ni1—N41—Ni1^ii^	102.1 (2)
C26—N21—Ni1	126.7 (4)	N43—N42—N41	176.7 (7)
			
C16—N11—C12—C13	1.5 (9)	C26—N21—C22—C12	−177.8 (5)
Ni1—N11—C12—C13	−178.4 (5)	Ni1—N21—C22—C12	3.7 (7)
C16—N11—C12—C22	−179.2 (5)	N11—C12—C22—N21	−3.1 (8)
Ni1—N11—C12—C22	1.0 (7)	C13—C12—C22—N21	176.2 (6)
N11—C12—C13—C14	−1.1 (10)	N11—C12—C22—C23	177.6 (6)
C22—C12—C13—C14	179.6 (6)	C13—C12—C22—C23	−3.1 (10)
C12—C13—C14—C15	−0.5 (10)	N21—C22—C23—C24	−0.7 (10)
C13—C14—C15—C16	1.7 (10)	C12—C22—C23—C24	178.6 (6)
C13—C14—C15—C17	−178.0 (7)	C22—C23—C24—C25	−1.6 (10)
C12—N11—C16—C15	−0.2 (10)	C23—C24—C25—C26	2.9 (10)
Ni1—N11—C16—C15	179.6 (5)	C23—C24—C25—C27	−175.4 (7)
C14—C15—C16—N11	−1.4 (11)	C22—N21—C26—C25	−0.1 (9)
C17—C15—C16—N11	178.3 (7)	Ni1—N21—C26—C25	178.2 (5)
C26—N21—C22—C23	1.6 (9)	C24—C25—C26—N21	−2.1 (10)
Ni1—N21—C22—C23	−177.0 (5)	C27—C25—C26—N21	176.2 (7)

Symmetry codes: (i) −*x*, −*y*+1, −*z*+1; (ii) −*x*+1, −*y*+1, −*z*+1.

### catena-Poly[[(5,5'-dimethyl-2,2'-bipyridine)nickel(II)]-µ2-azido-κ2N:N-µ2-azido-κ2N:N'] Hydrogen-bond geometry (Å, º)

**Table:**

*D*—H···*A*	*D*—H	H···*A*	*D*···*A*	*D*—H···*A*
C14—H14···N43^iii^	0.95	2.50	3.334 (9)	147
C17—H17*C*···N43^iv^	0.98	2.55	3.492 (11)	162

Symmetry codes: (iii) *x*+1, *y*, *z*+1; (iv) *x*+1/2, −*y*+3/2, *z*+1/2.

### Selected bond distances (Å)

**Table:**

Ni1—N11	2.045 (5)	Ni1—N41	2.090 (6)
Ni1—N21	2.074 (5)	Ni1—N33^i^	2.171 (7)
Ni1—N31	2.096 (5)	Ni1—N41^ii^	2.165 (6)
N31—N32	1.167 (7)	N41—N42	1.217 (7)
N32—N33	1.175 (8)	N42—N43	1.141 (8)

Symmetry codes: (i) -x, 1 - y, 1 - z; (ii) 1 - x, 1 - y, 1 - z.

### Hydrogen bond and short intermolecular contact parameters (Å, °)

**Table:**

D-H···A		D-H	H···A	D···A	D-H···A
C14-H14···N43^iii^		0.95	2.50	3.334 (9)	147
C17-H17C···N43^iv^		0.98	2.55	3.492 (11)	162

Symmetry codes: (iii) 1 + x, y, 1 + z; (iv) 0.5 + x, 1.5 - y, 0.5 + z.
